# Subcortical correlates of sleep in aging and Alzheimer’s disease: an *in vivo* study

**DOI:** 10.1093/braincomms/fcag360

**Published:** 2026-09-24

**Authors:** Agnès Pérez-Millan, Gerard Mayà, Isabel Martín-Sobrino, Marta Peña-González, Andrea Val-Guardiola, Guadalupe Fernández-Villullas, Beatriz Bosch, Adrià Tort-Merino, Albert Lladó, Manuel Morales-Ruiz, Christine M Walsh, Thomas C Neylan, Carles Gaig, Emma Muñoz-Moreno, Raquel Sánchez-Valle, Alex Iranzo, Lea T Grinberg, Neus Falgàs

**Affiliations:** Alzheimer’s Disease and Other Cognitive Disorders Unit, Hospital Clínic de Barcelona, Fundació de Recerca Clínic Barcelona-IDIBAPS, Barcelona CP08036, Catalonia, Spain; Centro de Investigación Biomédica en Red en Enfermedades Neurodegenerativas (CIBERNED), Madrid CP28029, Spain; eHealth Center, Faculty of Computer Science, Multimedia and Telecommunications, Universitat Oberta de Catalunya, Barcelona CP08018, Spain; Sleep Unit, Neurology Service, Hospital Clínic Universitari de Barcelona, University of Barcelona, Barcelona CP08036, Spain; Alzheimer’s Disease and Other Cognitive Disorders Unit, Hospital Clínic de Barcelona, Fundació de Recerca Clínic Barcelona-IDIBAPS, Barcelona CP08036, Catalonia, Spain; Magnetic Resonance Imaging Core Facility, Institut d’Investigacions Biomèdiques August Pi i Sunyer (IDIBAPS), Barcelona CP08036, Catalonia, Spain; Alzheimer’s Disease and Other Cognitive Disorders Unit, Hospital Clínic de Barcelona, Fundació de Recerca Clínic Barcelona-IDIBAPS, Barcelona CP08036, Catalonia, Spain; Alzheimer’s Disease and Other Cognitive Disorders Unit, Hospital Clínic de Barcelona, Fundació de Recerca Clínic Barcelona-IDIBAPS, Barcelona CP08036, Catalonia, Spain; Centro de Investigación Biomédica en Red en Enfermedades Neurodegenerativas (CIBERNED), Madrid CP28029, Spain; Alzheimer’s Disease and Other Cognitive Disorders Unit, Hospital Clínic de Barcelona, Fundació de Recerca Clínic Barcelona-IDIBAPS, Barcelona CP08036, Catalonia, Spain; Centro de Investigación Biomédica en Red en Enfermedades Neurodegenerativas (CIBERNED), Madrid CP28029, Spain; Alzheimer’s Disease and Other Cognitive Disorders Unit, Hospital Clínic de Barcelona, Fundació de Recerca Clínic Barcelona-IDIBAPS, Barcelona CP08036, Catalonia, Spain; Centro de Investigación Biomédica en Red en Enfermedades Neurodegenerativas (CIBERNED), Madrid CP28029, Spain; Alzheimer’s Disease and Other Cognitive Disorders Unit, Hospital Clínic de Barcelona, Fundació de Recerca Clínic Barcelona-IDIBAPS, Barcelona CP08036, Catalonia, Spain; Centro de Investigación Biomédica en Red en Enfermedades Neurodegenerativas (CIBERNED), Madrid CP28029, Spain; Biochemistry and Molecular Genetics Department-CDB, Hospital Clinic, IDIBAPS, CIBERehd, Barcelona CP08036, Spain; Department of Neurology, Fein Memory and Aging Center, Weill Institute for Neurosciences, University of California, San Francisco, CA 94143, USA; Department of Neurology, Fein Memory and Aging Center, Weill Institute for Neurosciences, University of California, San Francisco, CA 94143, USA; Department of Psychiatry, University of California, San Francisco, CA 94143, USA; San Francisco Veterinary Affairs Medical Center, San Francisco, CA 94121, USA; Sleep Unit, Neurology Service, Hospital Clínic Universitari de Barcelona, University of Barcelona, Barcelona CP08036, Spain; Magnetic Resonance Imaging Core Facility, Institut d’Investigacions Biomèdiques August Pi i Sunyer (IDIBAPS), Barcelona CP08036, Catalonia, Spain; Alzheimer’s Disease and Other Cognitive Disorders Unit, Hospital Clínic de Barcelona, Fundació de Recerca Clínic Barcelona-IDIBAPS, Barcelona CP08036, Catalonia, Spain; Centro de Investigación Biomédica en Red en Enfermedades Neurodegenerativas (CIBERNED), Madrid CP28029, Spain; Universitat de Barcelona, Barcelona CP08036, Spain; Sleep Unit, Neurology Service, Hospital Clínic Universitari de Barcelona, University of Barcelona, Barcelona CP08036, Spain; Department of Neuroscience, Mayo Clinic, Jacksonville, FL 32224, USA; Department of Laboratory Medicine and Pathology, Mayo Clinic, Jacksonville, FL 32224, USA; Department of Pathology, University of Sao Paulo Medical School, Sao Paulo 01246-903, Brazil; Alzheimer’s Disease and Other Cognitive Disorders Unit, Hospital Clínic de Barcelona, Fundació de Recerca Clínic Barcelona-IDIBAPS, Barcelona CP08036, Catalonia, Spain; Centro de Investigación Biomédica en Red en Enfermedades Neurodegenerativas (CIBERNED), Madrid CP28029, Spain

**Keywords:** Alzheimer’s disease, subcortical, locus coeruleus, magnetic resonance imaging, sleep

## Abstract

Sleep disturbances are frequently observed in patients with neurodegenerative diseases, but *in vivo* evidence linking subcortical sleep–wake structures to sleep phenotypes is limited. We aim to examine associations between MRI-derived measures of subcortical sleep–wake regulating regions and polysomnographic sleep parameters in individuals with Alzheimer’s disease and cognitively healthy controls. Fifty-seven adults (45 with biomarker-confirmed Alzheimer’s disease and 12 cognitively healthy controls with normal pTau217 plasma levels) were recruited at the Hospital Clínic de Barcelona. All participants completed overnight polysomnography and 3T brain MRI, including a neuromelanin-sensitive sequence. MRI-derived measures of wake-promoting structures [locus coeruleus (LC), posterior hypothalamus and basal forebrain] and sleep-promoting regions (anterior hypothalamus) were obtained. We estimated the association between nocturnal polysomnographic variables [including total sleep time, sleep efficiency, time in rapid eye movement (REM) and non-REM sleep stages, REM sleep latency, wake after sleep onset and arousals] and MRI-derived measures. Then, a principal component analysis (PCA) was used to obtain derived patterns of sleep–wake regulation. Among sleep-promoting regions, higher anterior hypothalamic volumes correlated with greater time in bed (*ρ* = 0.19, *P* < 0.05), increased sleep period time (SPT) (*ρ* = 0.25, *P* < 0.01), and decreased REM over SPT (*ρ* = −0.22, *P* < 0.05). Within wake-promoting structures, higher posterior hypothalamic volume and LC integrity were associated with higher arousals (*ρ* = 0.32, *P* < 0.05 and *ρ* = 0.18, *P* < 0.05, respectively), and higher basal forebrain volume was associated with less REM over SPT (*ρ* = −0.25, *P* < 0.05). Control anatomic regions showed no associations with sleep parameters. The first two principal components of the PCA explain 47.7% of the variance. Anterior hypothalamic volumes clustered with longer, more efficient sleep, whereas LC integrity aligned with increased wakefulness and REM sleep latency. Alzheimer’s disease participants showed a shift towards the latter pattern, indicating disrupted sleep–wake regulation. MRI-derived measures of subcortical sleep–wake regions were associated with clinical sleep phenotypes in Alzheimer’s disease and ageing. These *in vivo* findings align with prior post-mortem research, reinforcing the role of subcortical structures in sleep disturbances and highlighting these circuits as potential targets to alleviate sleep symptoms and modify disease progression.

## Teaser

Pérez-Millan *et al*. report that MRI markers of subcortical sleep–wake regions are associated with polysomnographic sleep patterns in ageing and Alzheimer’s disease. Anterior hypothalamic volume related to longer sleep, whereas locus coeruleus and posterior hypothalamic measures related to arousals, implicating subcortical circuits in sleep disruption.

## Graphical Abstract

**Graphical Abstract:**
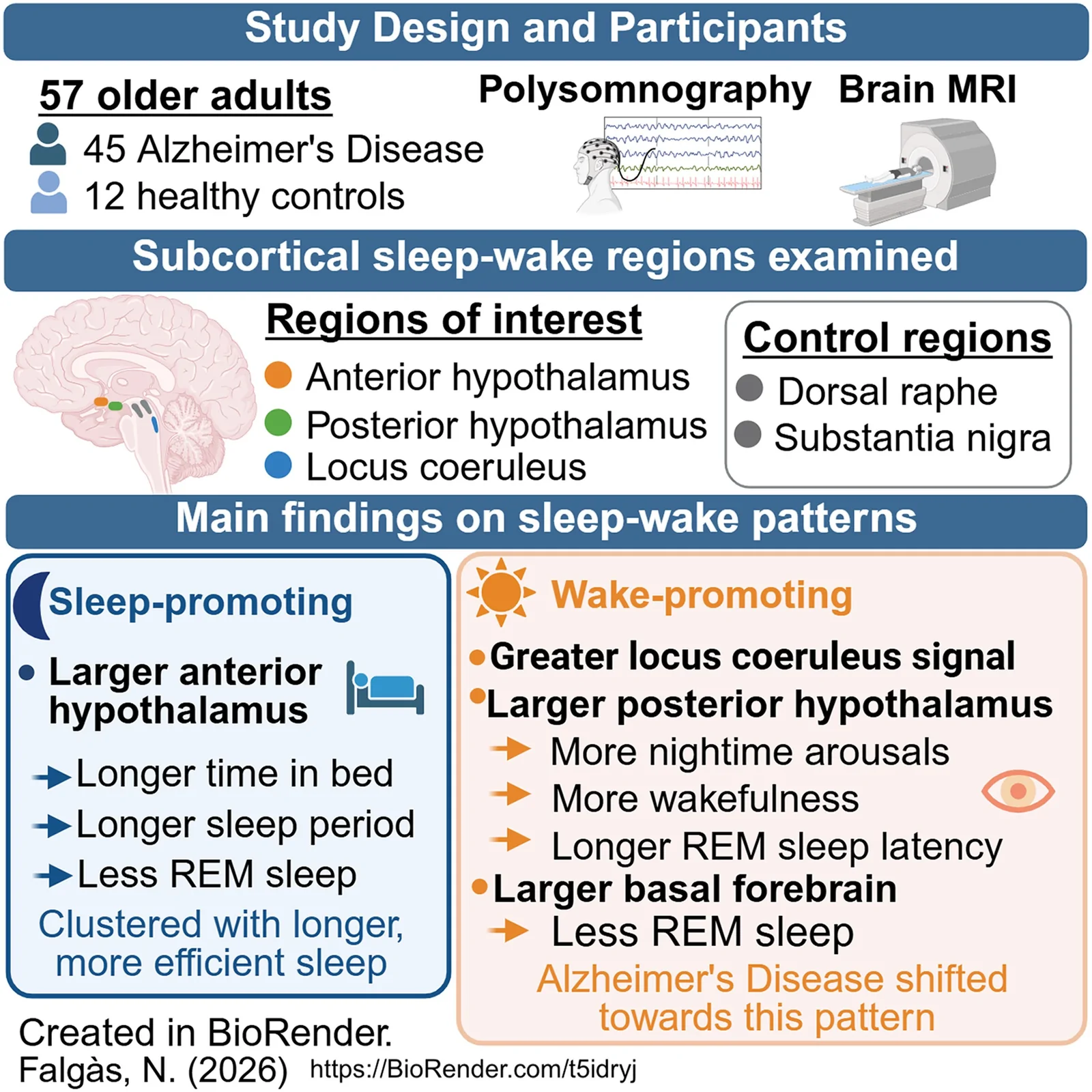

## Introduction

Sleep disturbances are highly prevalent in individuals with Alzheimer’s disease and often emerge early in the disease course, contributing to cognitive decline, behavioural symptoms, and reduced quality of life.^1-3^ While cortical pathology has been extensively studied, increasing evidence highlights the critical role of subcortical sleep–wake regulating structures—including the hypothalamus, locus coeruleus (LC), basal forebrain and brainstem nuclei—in maintaining normal sleep architecture.^4^ These nuclei are part of the so-called neuromodulatory subcortical systems, also known as the isodendritic core, a highly interconnected network that modulates higher brain functions, such as arousal, attention and sleep–wake transitions.^5^ Notably, these regions are among the earliest sites of tau pathology in Alzheimer’s disease, preceding cortical tau and β-amyloid deposition, as demonstrated by neuropathological studies.^6-9^ The degeneration of wake- andsleep-promoting neurons has been linked to excessive daytime sleepiness, sundowning and fragmented nocturnal sleep. Experimental models indicate that sleep disruption impairs essential cellular and extracellular processes, such as proteostasis, glymphatic clearance and redox homeostasis, while human studies show that sleep–wake cycles influence the accumulation and clearance of neurodegeneration-associated proteins.^10,11^ Conversely, deposition of these proteins can exacerbate sleep disruption, highlighting a bidirectional relationship between sleep disturbances and neurodegenerative processes.^12^

Our previous post-mortem study, including brain tissue from patients with Alzheimer’s disease and progressive supranuclear palsy (PSP) ,^13^ quantitatively demonstrated that higher neuronal counts in subcortical wake-promoting nuclei—including the LC, lateral hypothalamic area (LHA) and tuberomammillary nucleus (TMN)—were associated with lower homeostatic sleep drive, providing anatomical evidence for their contribution to clinical sleep phenotypes.^14-17^ However, post-mortem analyses are inherently limited in capturing dynamic *in vivo* relationships, leaving critical gaps in understanding whether these associations reflect ongoing disease processes. *In vivo* studies are therefore essential to investigate how subcortical structural integrity relates to objectively measured sleep patterns in living individuals and to validate post-mortem findings.^18^

Prior *in vivo* neuroimaging studies have begun to examine the structural and functional correlates of sleep–wake disruption within these subcortical systems. In Alzheimer’s disease, hypothalamic dysfunction measured with [18F]FDG-PET has been associated with sleep impairment and CSF Alzheimer’s disease biomarkers, supporting the relevance of hypothalamic alterations to clinical sleep phenotypes.^19^ More recent MRI studies have further linked hypothalamic subunit volumes with sleep quality and dementia risk factors in cognitively healthy adults.^20^ In parallel, neuromelanin-sensitive MRI studies of the LC have shown associations between LC integrity and nocturnal awakenings in the context of Alzheimer’s disease-related biomarker risk, consistent with the role of the LC–noradrenergic system in arousal regulation.^21^ Finally, basal forebrain MRI studies have reported associations between cholinergic basal forebrain volume and rapid eye movement (REM) sleep characteristics in individuals with amnestic mild cognitive impairment (MCI) .^22^ Together, these studies support the feasibility and relevance of studying sleep–wake regulating structures *in vivo*, but most have focused on single nuclei or self-reported sleep measures rather than jointly assessing multiple subcortical sleep–wake regions with objective polysomnography (PSG) in biomarker-defined Alzheimer’s disease.

In addition to structural MRI markers, fluid biomarkers of neuromodulatory systems may provide complementary information on sleep–wake regulation. Orexin/hypocretin neurons, located primarily in the lateral and posterior hypothalamus, are critical for maintaining wakefulness and stabilizing sleep–wake transitions, whereas noradrenaline is the principal neurotransmitter of the LC and plays a central role in arousal regulation. Therefore, CSF orexin and noradrenaline levels may serve as exploratory neurochemical markers related to the same hypothalamic and LC systems assessed structurally in this study.

To address this gap, the present study investigated the relationship between MRI-derived measures of subcortical sleep- and wake-promoting nuclei and PSG-assessed sleep parameters in individuals with biomarker-confirmed Alzheimer’s disease and cognitively healthy controls (CTR). Based on prior post-mortem and *in vivo* neuroimaging evidence, we hypothesized that preserved volumes of sleep-promoting regions, such as the anterior hypothalamus, would be associated with longer and more efficient sleep, whereas MRI-derived measures of wake-promoting regions, including the posterior hypothalamus, basal forebrain and LC, would be associated with greater wakefulness, sleep fragmentation and REM sleep-related alterations.^13^ In secondary exploratory analyses restricted to Alzheimer’s disease participants with available CSF samples, we also examined whether CSF orexin and noradrenaline levels were associated with polysomnographic sleep parameters, as complementary neurochemical markers of hypothalamic orexinergic and LC–noradrenergic arousal systems.

## Materials and methods

### Participants

The Hospital Clínic de Barcelona (HCB) Institutional Review Board approved the study, and all participants provided written informed consent (HCB/2021/0668). All participants were recruited from the Alzheimer’s Disease and Other Cognitive Disorders Unit at Hospital Clínic de Barcelona (Barcelona, Spain).

A summary of the study design is shown in Fig. 1.

**Figure 1:**
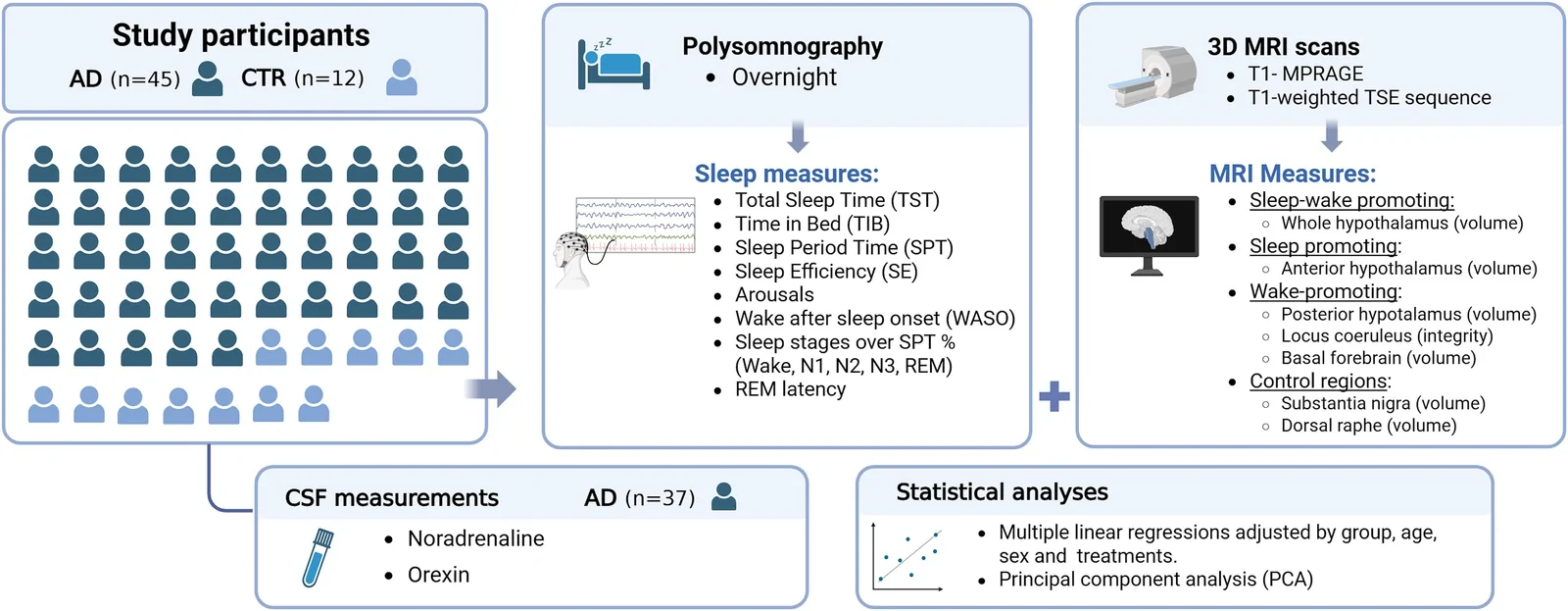
**Study flow diagram.** Created in BioRender. Falgas, N. (2026) https://BioRender.com/z0e3bw2.

We recruited a total of 57 participants classified as:

- Alzheimer’s disease (*n* = 45): mild cognitive impairment and mild dementia due to Alzheimer’s disease [clinical dementia rating (CDR) 0.5–1], meeting the criteria of biomarker-based Alzheimer’s disease diagnosis in agreement with the Alzheimer’s Association Workgroup diagnostic criteria.^19^ All Alzheimer’s disease participants were classified based on amyloid positivity, determined by CSF biomarkers or amyloid-PET.
- Healthy controls (CTR, *n* = 12): cognitive healthy individuals with no cognitive complaints, performance on the neuropsychological testing within the normal range (CDR 0) and biological confirmation of normal pTau217 plasma levels.

#### Inclusion and exclusion criteria

Participants were eligible if they were adults able to complete the study procedures, including overnight video-PSG and 3T brain MRI. Participants with major neurological disorders other than Alzheimer’s disease, major psychiatric disorders or medical conditions that could substantially interfere with study participation or interpretation of results were excluded. Sleep disorders, including insomnia, obstructive sleep apnoea and REM sleep behaviour disorder, were not used as a priori exclusion criteria in order to avoid selection bias and preserve the representativeness of sleep disturbances in ageing and Alzheimer’s disease.

### Sleep study

#### Polysomnography

All participants underwent overnight video-PSG performed at the Sleep Unit at the HCB. These studies consisted of vertical and horizontal electrooculography, electroencephalography (O2-A1, O1-A2, C4-A1, C3-A2, F4-A1, F3-A1) and surface EMG of the mentalis muscle, the right and left flexor digitorum superficialis muscles in the upper limbs and the right and left tibialis anterior muscles in the lower limbs. Additional measurements included electrocardiography, nasal and oral airflow with oral thermistors and nasal cannula, thoracic and abdominal respiratory effort, oxygen saturation assessed by a finger pulse oximeter, microphone and digital synchronized audiovisual monitoring. Sleep stages [percentage of wake after sleep onset (WASO), non-REM (NREM) N1, N2, N3, and REM] during the sleep period time (SPT) and the number of arousals were scored according to the American Academy of Sleep Medicine (AASM) guidelines.^20^ Total time in bed (TIB)—defined as the interval between lights off and lights on—differs from SPT, which refers specifically to the period between sleep onset and final awakening. NREM stands for non-rapid eye movement sleep, with stages N1, N2 and N3 representing progressively deeper levels of NREM sleep, while REM refers to rapid eye movement sleep.

### MRI study

#### MRI acquisition

All participants were scanned on a 3T Siemens PRISMA-FIT system using a 20-channel head coil at the Magnetic Resonance Imaging Core Facility, IDIBAPS, located at the Hospital Clínic de Barcelona. The MRI protocol included the acquisition of the following sequences:

- 3D T_1_-weighted magnetization-prepared rapid gradient-echo sequence (TR = 2300 ms, TE = 2.98 ms, TI = 900 ms, flip angle = 9°, bandwidth = 240 Hz/pixel, acquisition matrix = 256 × 256 × 240 and isometric voxel size=1 mm^3^), as a previously published paper of the Falgàs *et al*.^24^
- Neuromelanin-sensitive high-resolution, T_1_-weighted turbo spin-echo (TSE) sequence: aligned perpendicularly to the plane of the respective participant’s brainstem (acquisition time = 10.33 min) with the following parameters: TR = 600 ms, TE = 11 ms, flip angle=120°, bandwidth = 180 Hz/pixel, acquisition matrix = 320 × 320 × 16, voxel size = 0.5 × 0.5 × 1.8 mm^3^ and number of average = 7. TSE consists of 16 slices without gap, covering the pons.

#### Post-processing

- LC integrity: the acquired images were processed to estimate LC integrity as previously detailed by Falgàs *et al.*^24^ Three areas of interest were identified in the MNI template: including right and left LC as defined by the 2 standard deviation LC (2D-LC) map^25^ and a dorsal pontine reference region. Elastic registration between the MNI template and each participant’s T_1_-weighted image was performed to identify these three areas in each subject image. The integrity of LC was quantified according to the methodology described by Dahl *et al.*,^26^ namely, the ratio of the maximum intensity in the 2D-LC region to the reference area intensity averaged along the longitudinal axis.
- Neuromodulatory subcortical system volumes: volumes of hypothalamic and brainstem neuromodulatory nuclei were derived from automated segmentations in T_1_-weighted space using previously validated pipelines. Hypothalamic volumes (including the anterior and posterior hypothalamus) and basal forebrain volumes were obtained using Freesurfer (https://surfer.nmr.mgh.harvard.edu/) tools. Specifically, hypothalamic volume segmentation was performed using a convolutional neural network framework proposed by Billot *et al*.,^27^ while basal forebrain volumes were estimated using a deep learning toolbox described by Greve *et al*.^28^ Dorsal raphe nucleus and substantia nigra volumes were extracted by elastic registration between each subject’s T_1_-weighted image and the subcortical atlas described by Rolls *et al*.^29^ Segmentations were visually inspected for alignment and reviewed for accuracy prior to analysis.

### CSF measurements

See the Supplementary material for the CSF Alzheimer’s disease biomarker and plasma pTau217 methodologies.

#### CSF Noradrenaline

Noradrenaline was quantified using a plasma-HPLC kit (Chromsystems) after extraction from the CSF matrix via adsorption onto alumina.

#### CSF orexin

The concentration of orexin (hypocretin-1) was measured in CSF by radioimmunoassay (Phoenix Pharmaceuticals hypocretin-1 RIA kit) with intra- and inter-assay coefficients of variation of <5 and <20%, respectively.

### Statistical analyses

We compared demographic, clinical, sleep and MRI data across groups using the Wilcoxon rank-sum test for continuous variables and the Fisher exact test for discrete variables. Continuous variables were expressed as median and interquartile range (IQR: Q1, Q3).

Multiple linear regression analysis was used to evaluate the correlation between sleep and MRI variables. All models were adjusted for the clinical group, age, sex, and sleep treatments. Sleep treatments were defined as the use of medications with potential effects on sleep, including benzodiazepines such as lorazepam, trazodone, mirtazapine and quetiapine. Secondary exploratory analyses were conducted in Alzheimer’s disease participants with available CSF samples to examine associations between polysomnographic sleep measures and CSF orexin and noradrenaline levels.

We also analysed sleep and MRI variables with a principal component analysis (PCA), a dimensionality reduction method. The PCA is a multivariate method that maximizes variance. Thus, we performed the PCA to validate the relationship between the sleep and MRI measures. It was conducted to elucidate the key factors contributing to the highest variability in the dataset. First, all MRI and sleep variables were included in the PCA. Then, separate PCAs were also performed for only MRI and only sleep measures separately. In all PCAs, the variables were standardized by centering mean = 0 and scaling standard deviation = 1. By analysing the first principal component (PC) and the individual contributions of each variable to it, we estimated which variables accounted for the greatest variability in the data. Finally, biplots of the first and second PCs were used to assess the associations and directions among all variables.

We implemented the statistical analyses in R software (https://www.r-project.org) version 4.2.1. The significance level was set at *P* < 0.05 for all analyses. The code for these analyses is available at https://github.com/Agnes2/Sleep-Correlates/tree/main.

## Results

### Demographics and clinical data

Demographics and clinical data are detailed in Table 1. Patients with Alzheimer’s disease had lower MRI measures than CTR in all the studied subcortical regions (Table 1). The significant areas were the posterior and anterior hypothalamus (*P* = 0.02 and *P* < 0.001, respectively). No other MRI measures were significant, although Alzheimer’s disease showed lower overall mean compared to CTR. Of the 45 AD patients, 37 had reportable CSF levels of orexin [266.5 (251.1, 284.4) pg/mL] and noradrenaline [122.0 (58.0, 164.0) pg/mL], expressed as median and IQR (Q1, Q3).

**Table 1:** Demographics, clinical, sleep and MRI group summaries written as each measure’s median and IQR [Q1, Q3]

	All	CTR	Alzheimer’s disease	*P*
*N*	57	12	45	
Sex, men/women	27/30	7/5	20/25	0.4
Age, years (SD)	69.0 [63.0, 73.0]	59.5 [56.5,67.0]	70.0 [64.0,73.0]	**0.003**
Sleep treatments	14 (8%)	1 (8.3%)	13 (29%)	0.3
MMSE (SD)	25 [22, 28]	29 [28, 30]	25 [21, 27]	**<0**.**001**
*MRI measurements*				
LC integrity, adimensional	0.11 [0.10, 0.13]	0.12 [0.10, 0.13]	0.11 [0.10,0.13]	0.9
Posterior hypothalamus, mm^3^	116.6 [103.8, 123.3]	121.8 [111.8, 127.0]	110.4 [101.0, 120.5]	**0**.**02**
Anterior hypothalamus, mm^3^	21.1 [18.6, 25.3]	25.9 [23.6, 26.8]	20.4 [17.8, 23.8]	**<0**.**001**
Whole hypothalamus, mm^3^	394.8 [371.2, 422.5]	405.4 [394.8, 428.6]	385.3 [361.2, 410.0]	0.09
Dorsal raphe, mm^3^	109.0 [102.0, 121.0]	111.0 [104.0, 117.5]	108.0 [101.0, 122.0]	0.7
Basal forebrain, mm^3^	298.6 [269.1, 319.9]	312.5 [282.0, 335.8]	285.7 [263.1, 319.8]	0.12
SN pars compacta, mm^3^	172.0 [152.5, 192.0]	179.0 [154.5, 196.0]	171.0 [152.5, 189.0]	0.6
SN pars reticulata, mm^3^	313.0 [276.0, 347.0]	306.5 [264.0, 337.8]	319.0 [281.0, 347.0]	0.8
*Sleep disorders*				
Apnoea–hypopnoea index				0.6
Normal (<5)	14 (25%)	3 (25%)	11 (24%)	
Mild (5–15)	19 (33%)	6 (50%)	13 (29%)	
Moderate (16–30)	13 (23%)	2 (17%)	11 (24%)	
Severe (>30)	11 (19%)	1 (8.3%)	10 (22%)	
Insomnia	12 (21%)	0	12 (27%)	0.053
REM sleep behaviour disorder	7 (12%)	0	7 (16%)	0.3
*Sleep measurements*				
TST (min)	361.0 [300.0, 400.0]	386.5 [334.5, 427.5]	361.0 [259.0, 396.0]	0.2
TIB (min)	484.0 [460.0, 502.0]	483.0 [455.5, 498.0]	484.0 [465.0, 505.0]	0.4
SPT (min)	452.0 [431.0, 480.0]	450.5 [432.5, 469.0]	454.0 [431.0, 483.0]	0.9
Sleep efficiency, (%)	74.4 [62.6, 83.5]	77.3 [73.2, 89.4]	73.7 [56.2, 83.1]	0.07
Arousals, (count)	100.0 [66.0, 131.0]	109.0 [83.5, 148.0]	87.0 [61.0, 130.0]	0.3
WASO, min	82.5 [52.5, 141.5]	64.5 [27.8, 99.0]	90.0 [61.0, 144.0]	0.09
N1 over SPT (%)	12.3 [8.2, 17.6]	13.1 [7.0, 14.7]	11.9 [8.6, 18.1]	0.5
N2 over SPT (%)	38.8 [30.1, 48.0]	45.9 [32.4, 51.7]	38.8 [29.7, 46.7]	0.3
N3 over SPT (%)	11.3 [2.5, 48.0]	13.3 [9.5, 21.3]	10.8 [2.2, 15.1]	0.08
Wake over SPT (%)	19.1 [12.8, 30.2]	15.9 [6.1, 21.8]	21.2 [12.9, 36.9]	0.09
REM over SPT (%)	13.8 [8.9, 17.4]	15.5 [12.8, 19.3]	13.4 [8.3, 17.3]	0.1
REM sleep latency over SPT, min	106.5 [72.5, 217.0]	94.3 [77.0, 139.0]	108.0 [68.0, 230.0]	0.6

We calculated differences between groups using the Fisher test for sex and the Wilcoxon rank-sum test for the remaining variables. We highlighted the significant group differences in bold. Specific sleep treatments were lorazepam, quetiapine, trazodone and mirtazapine.

### Sleep, MRI and CSF analysis

To maximize statistical power and capture the full spectrum of variability across participants, we performed continuous analyses combining all subjects rather than restricting comparisons to diagnostic groups. First, we examine the correlation between MRI subcortical measures and clinical sleep measurements (Fig. 2). Greater whole hypothalamus volume (sleep- and wake-promoting region) correlated with high arousals (*r* = 0.43, *P* = 0.01). The sleep-promoting region, the anterior hypothalamus, was associated with greater TIB (*r* = 0.19, *P* = 0.03) and SPT (*r* = 0.25, *P* = 0.001), and with less REM % over SPT (*r* = –0.22, *P* = 0.02). Regarding wake-promoting regions, the posterior hypothalamic volume was positively correlated with SPT (*r* = 0.18, *P* = 0.03) and with arousals (*r* = 0.32, *P* = 0.03). Higher LC integrity was also associated with more arousals (*r* = 0.18, *P* = 0.02). In contrast, basal forebrain volume showed a negative correlation with REM over SPT (*r* = −0.25, *P* = 0.03). Finally, the substantia nigra pars reticulata, substantia nigra pars compacta and the dorsal raphe, which were included as control regions, did not show any significant correlations. See Supplementary Fig. 1 for the analysis corrected by apnoea with similar results.

**Figure 2:**
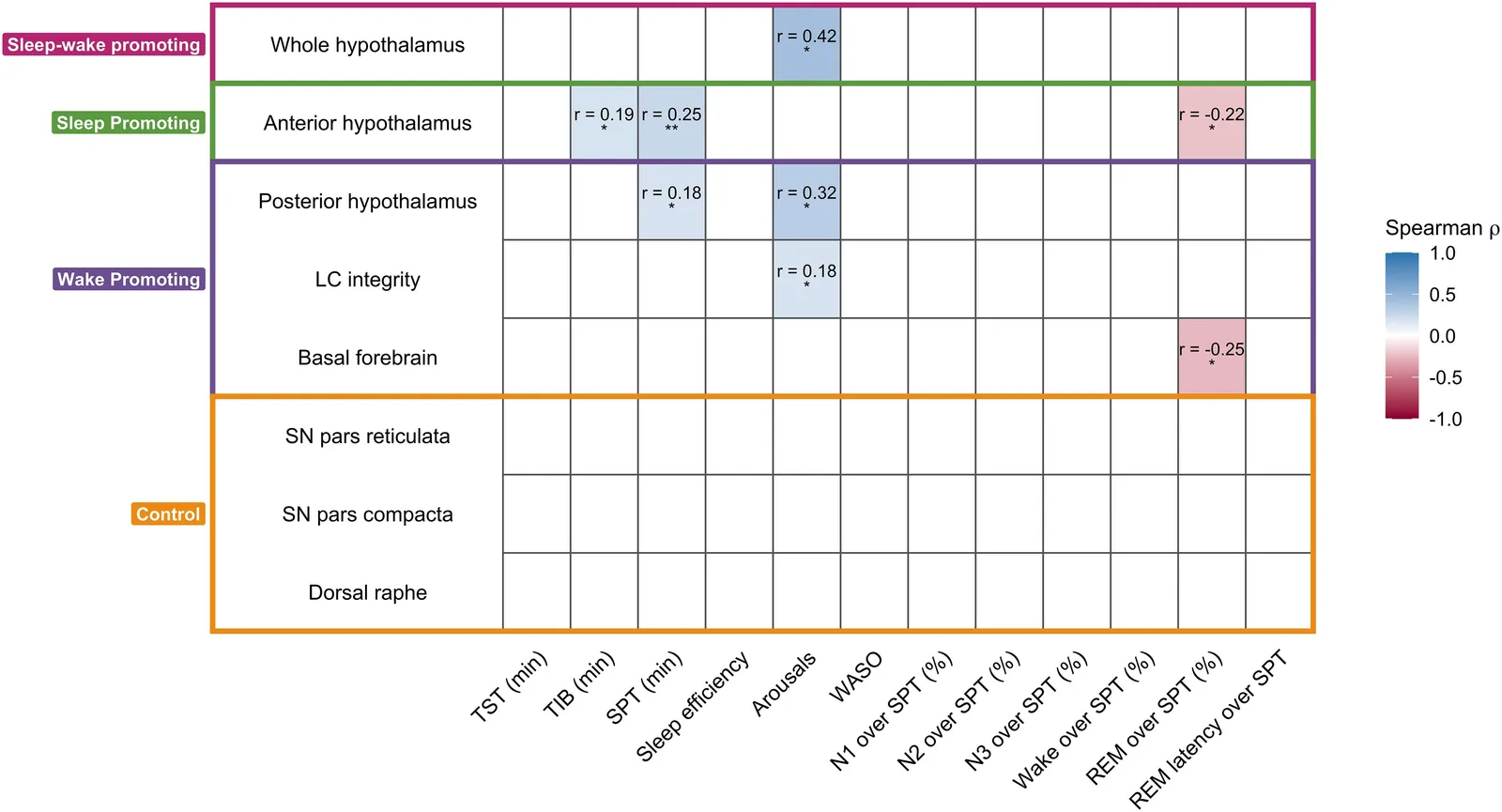
**Correlation between subcortical MRI regions and clinical sleep measurements for all the participants.** Dark blue indicates a stronger positive correlation, and darker red indicates a stronger negative correlation. It only shows the significant correlations. Symbols indicate significance levels: *P-value < 0.05 and **P-value < 0.01. Substantia nigra (SN).

Second, we examined the associations between CSF variables (orexin and noradrenaline) and clinical sleep parameters. We found significant positive correlations between orexin and both TIB (*r* = 0.40, *P* < 0.001) and arousals (*r* = 0.26, *P* = 0.02), as well as between noradrenaline and REM over SPT (*r* = 0.61, *P* < 0.001). In analyses corrected for apnoea, results remained similar, and an additional positive correlation emerged between noradrenaline and N3 over SPT (*r* = 0.25, *P* < 0.05). See Supplementary Fig. 1 for further details.

### Principal component analysis

The PCA was conducted to confirm the correlation between sleep and MRI measurements. The first PC reflects the dominant pattern of the main variance. It showed that sleep variables captured greater variance compared to MRI variables. The composition of the first PC (Fig. 3A) of the five most important variables included total sleep time (TST) (14.84%), sleep efficiency (14.02%), wake over SPT (11.07%), WASO (7.90%) and SPT (7.56%). In brief, WASO represents the total minutes awake after sleep onset, whereas wake over SPT expresses wakefulness as a percentage of the total SPT.

**Figure 3:**
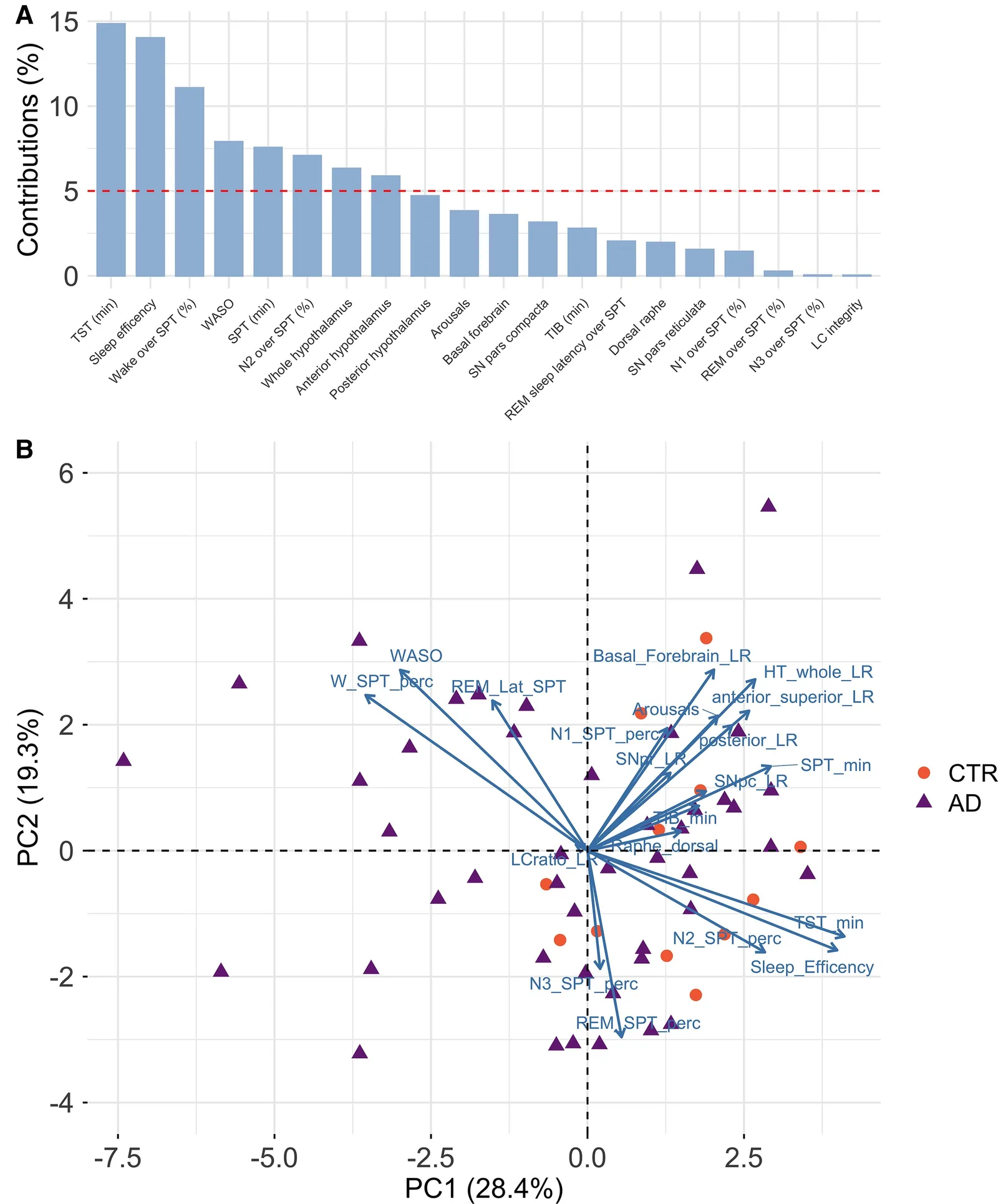
**PCA results for all the data with subcortical MRI and clinical sleep parameters.** (**A**) Results of the first PC of the most contributing variables. The dashed red indicates the average contribution. A variable with a contribution greater than this limit is important to the PC. (**B**) Biplot of the first and second PCs (eigenvalues 5.68 and 3.85, respectively) shows individual samples coloured and shaped according to their diagnosis (CTR or Alzheimer’s disease). The plot illustrates group separation along the first and second PCs and highlights the variables with the strongest influence (indicated by the larger arrows). The length and direction of the arrows indicate the strength and orientation of each variable’s contribution: variables pointing in similar directions are positively correlated, whereas those pointing in opposite directions show negative correlations. Substantia nigra (SN).

The PCA biplot (Fig. 3B) illustrates the loadings of sleep and MRI variables on the first and second PC. The biplot emphasizes the variables that have the greatest impact, illustrating both the magnitude and direction of each variable’s contribution. Variables that point in similar directions are positively correlated, while those pointing in opposite directions indicate negative correlations. The first PC (28.4%) reflected a continuum of consolidated sleep variables, characterized by higher values, except for WASO, wake over SPT, REM sleep latency over SPT and LC integrity. See Supplementary Figs 2 and 3, the PCA for only MRI or sleep measures. The second PC (19.3%) distinguished variables related to all MRI variables and most sleep variables, except TST, sleep efficiency, REM over SPT and N2 and N3 over SPT. The distributions of CTR and Alzheimer’s disease participants show partial overlap, yet they reveal meaningful relationships among sleep and MRI measures. However, the variables WASO, wake over SPT, REM sleep latency over SPT, and LC integrity show the loadings oriented towards the region predominantly occupied by Alzheimer’s disease individuals.

## Discussion

In this cross-sectional MRI–PSG study of biomarker-confirmed Alzheimer’s disease and cognitively unimpaired older adults, we found that the subcortical sleep–wake nuclei relate to objectively measured nocturnal sleep. Higher anterior hypothalamic volume was associated with longer nocturnal sleep and a lower proportion of REM sleep across the night. In contrast, larger posterior hypothalamic volume and greater LC integrity both tracked with increased nocturnal arousals, suggesting a tendency towards more fragmented sleep. Basal forebrain volume showed an inverse association with REM sleep proportion, while control anatomic regions such as the dorsal raphe and substantia nigra were not significantly related to any sleep parameters. Notably, none of the subcortical measures showed significant univariate associations with REM sleep latency, although REM sleep latency clustered with LC integrity and fragmentation indices in the PCA, aligned with the region of the biplot predominantly occupied by individuals with Alzheimer’s disease.

These *in vivo* findings extend and complement our previous clinicopathological study, in which, we combined PSG or EEG with quantitative post-mortem stereology of noradrenergic LC, orexinergic LHA and histaminergic TMN neurons in patients with Alzheimer’s disease, PSP and healthy controls.^13^ In that work, higher numbers of wake-promoting neurons were associated with decreased homeostatic sleep drive as well as longer REM sleep latency, and k-means clustering/PCA identified an ‘Alzheimer’s disease-predominant’ pattern with relatively high sleep drive and a ‘PSP-predominant’ pattern characterized by hyperarousal and pronounced insomnia. The current MRI study recapitulates several key aspects of this organization in living individuals: imaging markers of wake-promoting nodes (the posterior hypothalamus, basal forebrain and LC) again align with a wake-biased, sleep-fragmented phenotype, whereas the anterior hypothalamus—primarily encompassing sleep-promoting areas, such as the ventrolateral preoptic area and median preoptic nucleus—is associated with metrics of longer sleep.

At the whole-structure level, larger hypothalamic volume was associated with a higher number of nocturnal arousals, but subregional analyses revealed more physiologically coherent, divergent patterns. Anterior hypothalamic volume is linked to longer TIB and SPT and to a lower proportion of REM sleep, consistent with a stronger non-REM sleep drive and the inhibitory role of preoptic GABAergic neurons on wake-promoting systems.^30^ Imaging and post-mortem studies in Alzheimer’s disease demonstrate that degeneration in these anterior regions correlates with impaired sleep efficiency and reduced REM sleep.^19,20,31^ In contrast, the posterior hypothalamus, which contains wake-promoting LHA and TMN, correlated positively with arousals and SPT, consistent with prolonged but fragmented sleep, and preservation of these neurons has previously been linked to decreased sleep drive.^13,32^ Together, these findings suggest that the sleep phenotype reflects a balance between degeneration and relative preservation of hypothalamic subnuclei with opposing effects on sleep–wake regulation, while highlighting the limitations of macroanatomical hypothalamic segmentations, which may average across functionally distinct cell groups.

A similar issue of macrostructural averaging likely also applies to our findings in the basal forebrain. Despite the well-established wake- and REM-promoting role of the nucleus basalis of Meynert (NbM), we observed an inverse association between basal forebrain volume and proportion of REM sleep, in contrast to prior *in vivo* data reporting a positive association between NbM volume and REM sleep in amnestic MCI.^4^ This discrepancy may reflect methodological and anatomical differences: while André *et al*.^22^ targeted a more precise NbM delineation, our analyses encompassed a broader basal forebrain region comprising heterogeneous neuronal populations with distinct and even opposing roles in sleep–wake control. In animal models, cholinergic, glutamatergic and PV + GABAergic neurons within the basal forebrain are wake/REM-active, whereas SOM + GABAergic neurons promote NREM sleep, underscoring this cellular heterogeneity^33^. Furthermore, the NbM is among the earliest and most severely affected nuclei in Alzheimer’s disease, with marked neuronal loss and tau pathology across neuropathological subtypes.

Our LC findings are consistent with this interpretation of wake-promoting nuclei as contributors to sleep fragmentation in early Alzheimer’s disease.^21,34^ In our cohort, higher LC integrity was associated with a greater number of nocturnal arousals, and in the PCA, LC loadings clustered with WASO, wake over SPT and REM sleep latency over SPT towards the region of the biplot predominantly occupied by Alzheimer’s disease participants. This pattern aligns with the role of the LC as a central noradrenergic hub that promotes cortical activation and stabilizes wakefulness, as supported by animal studies showing that LC tonic firing increases during wake and suppresses REM and NREM sleep, and that optogenetic activation of LC neurons fragments sleep.^35-37^ Neuropathological and *in vivo* imaging studies further indicate that the LC is one of the earliest sites of tau deposition in Alzheimer’s disease and that reduced LC integrity is associated with cognitive impairment, neuropsychiatric symptoms and altered arousal regulation.^24,38-40^ In our previous post-mortem work, higher LC neuronal counts were linked to reduced sleep drive and an insomnia-like phenotype across Alzheimer’s disease and PSP, including longer REM sleep latency, suggesting that relative preservation of LC neurons, particularly in early disease stages, maintains a hyperarousal state and contributes to fragmented and altered REM timing in ageing and Alzheimer’s disease, through interactions with hypothalamic and basal forebrain circuits.^13^

We did not observe significant associations between MRI-derived subcortical measures and N3 sleep, expressed as percentage over SPT. This should be interpreted cautiously because the present study focused on conventional PSG-derived sleep macroarchitecture rather than EEG microstructural measures. Although slow-wave sleep is closely linked to restorative sleep physiology, CSF dynamics, glymphatic clearance and Alzheimer’s disease-related protein homeostasis, these mechanisms may be more directly reflected by quantitative EEG features than by N3% alone.^41-43^ Indeed, N3% does not distinguish between different components of NREM slow-wave physiology, including slow oscillation power, delta power, slow-wave density or wave amplitude. This distinction is relevant because human neuroimaging and animal studies have shown that neural slow waves during NREM sleep are coupled to haemodynamic oscillations and CSF flow, supporting a mechanistic link between slow oscillatory activity and brain clearance.^37,44^ In our recent study specifically designed to address this question, we examined LC integrity, quantitative slow-wave sleep measures and MRI-visible perivascular spaces as an indirect marker related to glymphatic/clearance dysfunction across ageing and Alzheimer’s disease.^23^ That study showed that LC integrity and perivascular space burden were associated with slow-wave sleep spectral measures, particularly slow oscillatory activity, rather than with conventional sleep-stage proportions. Thus, the present null finding for N3% should not be interpreted as evidence against a role of subcortical arousal systems in slow-wave physiology but rather as reflecting the macroarchitectural scope of the current analysis.

We also examined fluid biomarkers of key neuromodulators by measuring CSF orexin and noradrenaline in the Alzheimer’s disease group. Higher orexin levels were associated with longer TIB and more arousals in keeping with its wake-promoting role.^19,31^ The finding of higher noradrenaline being associated with a greater proportion of REM over SPT was unexpected. In addition, in supplementary analyses, higher noradrenaline levels were positively associated with N3 sleep percentage over SPT. Notably, this association was observed for CSF noradrenaline but not for MRI-derived measures of LC integrity, which showed no significant relationship with N3 sleep. This dissociation suggests that functional noradrenergic tone may be more closely linked to slow-wave sleep macroarchitecture than structural LC measures in the present dataset, although these findings warrant further investigation.

In this relatively small, single-centre cohort (12 cognitively unimpaired controls and 45 amyloid-positive participants, most in the MCI stage), the study was not powered for formal between-group comparisons of sleep, and we did not detect statistically significant differences in conventional polysomnographic measures between Alzheimer’s disease and CTR. This contrasts with prior work in larger cohorts, which has consistently reported more fragmented and less restorative sleep along the Alzheimer’s disease continuum compared with cognitively healthy older adults.^45-47^ Thus, based on our data, we cannot confirm group-level sleep differences, but within-group associations between subcortical structure, neuromodulatory biomarkers and specific sleep parameters support the view that subcortical sleep–wake systems shape sleep regulation in a transdiagnostic manner in late life, rather than mapping cleanly onto diagnostic categories in a sample of this size. These findings do not rule out early involvement of these structures in Alzheimer’s disease; rather, they suggest that in this early-stage and relatively small cohort, sleep–wake alterations may be more detectable as continuous structure–function associations than as categorical Alzheimer’s disease versus control differences.

Several limitations should be considered when interpreting these results. First, our control group sample size may constrain generalizability and limit statistical power for between-group comparisons of sleep outcomes. Second, the cross-sectional design does not allow causal inferences. Third, our MRI metrics provide macrostructural proxies rather than direct measures of neuronal number or neurotransmitter function, and our segmentations do not resolve individual subnuclei or specific cell populations. Fourth, because we examined several sleep–MRI associations in an exploratory framework, the results should be confirmed in larger, independent cohorts. Moreover, CSF noradrenaline was quantified using an HPLC kit originally validated for plasma, which may be suboptimal for CSF quantification given matrix-specific differences. Future studies should validate these findings in larger cohorts including cognitively unimpaired controls and using assays specifically optimized for CSF noradrenaline measurement. Nevertheless, to our knowledge, this is the first *in vivo* study to combine high-resolution MRI of multiple subcortical sleep–wake nuclei with PSG in biomarker-defined Alzheimer’s disease.

## Conclusion

This study, together with our prior clinicopathological work, supports a central role of neuromodulatory subcortical systems in shaping sleep phenotypes across ageing and Alzheimer’s disease. Our *in vivo* MRI–PSG data, aligned with post-mortem findings, suggest that an imbalance between sleep- and wake-promoting nuclei underlies sleep disruption in ageing and Alzheimer’s disease and highlight these circuits as potential targets to alleviate sleep symptoms and modify disease progression.

## Supplementary Material

fcag360_Supplementary_Data

## Data Availability

Anonymized data can be shared upon request to the corresponding or senior author. However, data are likely to be subject to a data transfer agreement with restrictions required to comply with participant consent and data protection regulations. The code for these analyses is available at https://github.com/Agnes2/Sleep-Correlates/tree/main
