# Supplementary material for: Subcortical correlates of sleep in aging and Alzheimer’s disease: an in vivo study
Source: Brain Commun. 2026 Sep 24;8(5):fcag360. doi: 10.1093/braincomms/fcag360 (PMC13639700; doi:10.1093/braincomms/fcag360)
Supplement: Supplementary file 1 [file fcag360_supplementary_data.docx]

# Supplementary Material

**Subcortical correlates of sleep in aging and Alzheimer’s disease: an in vivo study**

Agnès Pérez-Millan, Gerard Mayà, Isabel Martín-Sobrino, Marta Peña-González, Andrea Val-Guardiola, Guadalupe Fernández-Villullas, Beatriz Bosch, Adrià Tort-Merino, Albert Lladó, Manuel Morales-Ruiz, Christine Walsh, Thomas C Neylan, Carles Gaig, Emma Muñoz-Moreno, Raquel Sánchez-Valle, Alex Iranzo, Lea T Grinberg, Neus Falgàs

**CSF measurements:**

*AD biomarkers:*

Lumbar punctures were performed in the morning to collect CSF samples for diagnostic purposes. Levels of CSF amyloid-β (Aβ42), total tau (T-tau), and phosphorylated tau (P-tau) were measured using Lumipulse G ELISAs, following the manufacturer's instructions (Fujirebio, Ghent, Belgium). Each CSF biomarker's cut-off values for abnormality were defined based on internal controls: Aβ42 ≤ 600 pg/ml, T-tau > 385 pg/ml, and P-tau > 65 pg/ml.

**Plasma biomarkers:**

*Plasma pTau 217:*

Blood samples were obtained at the first visit, centrifuged to obtain plasma, aliquoted, and stored at -80ºC. Plasma p-tau217 levels were determined with a commercially available Lumipulse G pTau 217 (Fujirebio, Japan) assay.

**Sleep, MRI and CSF Analysis considering apnea**

We examine the correlation between MRI subcortical measures and clinical sleep measurements (Supplementary Figure 1).

***Supplementary Figure 1:*** *Correlation between subcortical MRI regions and clinical sleep measurements for all the participants. Dark blue indicates stronger positive correlation and darker red a stronger negative correlation. It only shows the significant correlations. Symbols indicate significance levels: * p-value < 0.05 and ** p-value < 0.01.*

We found significant positive correlations between orexin and both time in bed (TIB, r=0.40, p<0.001) and arousals (r=0.26, p<0.01), as well as a between noradrenaline and REM over SPT (r=0.61, p<0.001) and N3 over SPT (r=0.25, p<0.05).

**Principal Component Analysis for Subcortical MRI Variables**

***Supplementary Figure 2:*** *Principal component analysis results for all the data with subcortical MRI parameters. A) Results of the first principal component of the most contributing variables. The dashed red indicates the average contribution. A variable with a contribution greater than this limit is important to the principal component.* *B) Biplot of the first and second principal components (eigenvalues 3.47 and 1.51 respectively) shows individual samples colored and shaped according to their diagnosis (CTR or AD). The plot illustrates group separation along the first and second principal components and highlights the variables with the strongest influence (indicated by the larger arrows).*

**Principal Component Analysis for Clinical Sleep Variables**

***Supplementary Figure 3:*** *Principal component analysis results for all the data with clinical sleep parameters. A) Results of the first principal component of the most contributing variables. The dashed red indicates the average contribution. A variable with a contribution greater than this limit is important to the principal component.* *B) Biplot of the first and second principal components (eigenvalues 4.97 and 2.72 respectively) shows individual samples colored and shaped according to their diagnosis (CTR or AD). The plot illustrates group separation along the first and second principal components and highlights the variables with the strongest influence (indicated by the larger arrows).*
